# Association between zinc deficiency and adverse outcomes in patients with dementia: a matched cohort study

**DOI:** 10.3389/fnut.2026.1801558

**Published:** 2026-03-20

**Authors:** I-Wen Chen, Li-Chen Chang, Yi-Chen Lai, Kuo-Chuan Hung

**Affiliations:** 1 Department of Anesthesiology, Chi Mei Medical Center, Liouying, Tainan City, Taiwan; 2 Department of Anesthesiology, E-Da Hospital, I-Shou University, Kaohsiung City, Taiwan; 3 Department of Anesthesiology, Chi Mei Medical Center, Tainan City, Taiwan

**Keywords:** dementia, mortality, prognostic marker, propensity score matching, sepsis, zinc deficiency

## Abstract

**Background:**

Zinc deficiency is prevalent among older adults and individuals with dementia; however, its prognostic significance in this population remains unclear. We investigated the association between zinc deficiency and clinical outcomes in patients with dementia.

**Methods:**

This retrospective matched cohort study used data from the TriNetX Research Network. Adult patients with dementia diagnosed between January 2010 and December 2024 were categorized according to their first recorded serum zinc measurement as zinc-deficient (<70 μg/dL) or normal (70–120 μg/dL). The date of the first qualifying zinc measurement served as the index date. The primary outcome was 1-year all-cause mortality. Secondary outcomes included sepsis, intensive care unit admission, urinary tract infection, pneumonia, acute kidney injury, and elevated C-reactive protein levels. Sensitivity, subgroup, and dose–response analyses were performed.

**Results:**

After matching, 1,241 patients remained in each cohort. Zinc deficiency was significantly associated with higher 1-year mortality (10.1% vs. 6.8%; hazard ratio [HR] 1.54; *p* = 0.002). Increased risks were also observed for sepsis (HR 1.76; *p* = 0.001), intensive care unit admission (HR 1.67; *p* = 0.004), urinary tract infection (HR 1.47; *p* < 0.001), and elevated C-reactive protein levels (HR 1.49; *p* = 0.002). These associations were attenuated during extended follow-up (1–3 years) and were no longer statistically significant. Dose–response analysis revealed that severe zinc deficiency (<50 μg/dL) conferred a greater mortality risk (HR 1.85; *p* = 0.002) and nearly three-fold higher sepsis risk (HR 2.92; *p* < 0.001). Sensitivity analyses confirmed the robustness of the findings.

**Conclusion:**

In this observational study, zinc deficiency was associated with increased 1-year mortality and infection-related complications in patients with dementia, with evidence of a dose–response relationship. However, these findings do not establish causation. Future prospective studies are warranted to determine whether zinc supplementation can improve the clinical outcomes of zinc-deficient patients with dementia.

## 1 Introduction

Dementia represents a major global health challenge, affecting approximately 55 million individuals worldwide, with projections indicating that the burden will triple by 2050 (1). Beyond progressive cognitive decline, patients with dementia face substantially elevated risks of infections, systemic inflammation, and other medical complications that contribute to excess mortality (2–6). Identifying prognostic factors associated with long-term outcomes in patients with dementia may facilitate the early recognition of high-risk individuals and guide clinical decision-making regarding monitoring intensity and preventive interventions (7–9). Zinc is an essential trace element that plays a critical role in immune function, antioxidant defense, and inflammatory regulation (10–12). Zinc deficiency impairs both innate and adaptive immunity, increases susceptibility to infections, and promotes systemic inflammation through enhanced activation of the NLRP3 inflammasome pathway (13–15). Notably, zinc deficiency is common among older adults and those in institutional care settings, and a substantial proportion of individuals with dementia exhibit suboptimal zinc status, reflecting the combined effects of age-related changes in intake, absorption, and metabolism, as well as increased physiological demands (16, 17).

Accumulating evidence supports the prognostic value of zinc status in various clinical populations. In critically ill patients, low serum zinc concentrations have been associated with greater illness severity and adverse outcomes, including higher organ dysfunction scores and mortality (18). In sepsis, persistently low serum zinc levels have been linked to recurrent septic episodes and worse survival (19). Among hospitalized medical patients, decreased plasma zinc concentrations independently predict long-term mortality, even after adjustment for validated severity/risk scores (20). These observations suggest that zinc deficiency may serve as a clinically meaningful prognostic marker in diverse patient populations.

Despite growing evidence linking zinc deficiency to adverse outcomes in other populations, its prognostic significance in patients with established dementia has not been well characterized. Dementia is frequently accompanied by nutritional inadequacy and increased vulnerability to infectious and inflammatory complications. Therefore, evaluating zinc status may provide clinically relevant prognostic information and help identify patients who could benefit from focused nutritional assessment. Based on this rationale, we hypothesized that zinc deficiency is independently associated with increased mortality and higher rates of infection-related complications in patients with dementia. To test these hypotheses, we conducted a retrospective matched cohort study using a large multicenter healthcare database to investigate the association between zinc deficiency and clinical outcomes, including all-cause mortality, sepsis, pneumonia, urinary tract infection, and systemic inflammation in patients with dementia.

## 2 Methods

### 2.1 Study design and data source

This retrospective matched cohort study was performed using data from the TriNetX Research Network, a large federated platform that aggregates de-identified electronic health records from a wide range of healthcare organizations throughout the United States. The database provides longitudinal patient-level information encompassing demographics, clinical diagnoses, procedures, medication prescriptions, and laboratory measurements provided by both academic medical centers and community healthcare systems. The study protocol was reviewed and approved by the Institutional Review Board of Chi Mei Medical Center, which waived the requirement for informed consent because the analysis was conducted exclusively on anonymized retrospective data.

### 2.2 Inclusion criteria

Adult patients (aged ≥18 years) with a diagnosis of dementia were identified between January 1, 2010, and December 31, 2024. Dementia was defined using the International Classification of Diseases, 10th Revision, Clinical Modification (ICD-10-CM) codes, including Alzheimer’s disease (G30), vascular dementia (F01), dementia in other diseases classified elsewhere (F02), and unspecified dementia (F03).

Patients were categorized into two exposure groups according to their first serum zinc level recorded during the study period. The zinc-deficient cohort comprised patients with zinc concentrations <70 μg/dL, whereas the control cohort included patients with zinc levels within the reference range (70–120 μg/dL). The reference range of 70–120 μg/dL was based on established clinical laboratory standards and international clinical chemistry references (21). The date of the first qualifying zinc measurement served as the index date for the cohort entry. Although zinc testing in clinical practice is not performed randomly and may be influenced by underlying clinical conditions, our comparison was restricted to patients who had undergone zinc measurement, ensuring that both groups were subjected to similar indications for testing.

### 2.3 Exclusion criteria

Several exclusion criteria were applied to minimize confounding and reverse causality. Patients were excluded if they had conditions known to substantially alter zinc metabolism or prognosis, including human immunodeficiency virus infection, prior bariatric surgery, end-stage renal disease, stage 4 or 5 chronic kidney disease, or a dependence on renal replacement therapy. To reduce bias from acute systemic illness, patients with diagnoses of acute kidney injury, sepsis, or severe sepsis, as well as those who received critical care services within 1 month before or after the index zinc measurement, were excluded. Patients with zinc concentrations above the upper reference limit (>120 μg/dL) were also excluded to ensure that the control group had a physiologically normal zinc status. Additionally, patients who died within 30 days of zinc measurement were excluded to minimize reverse causality from acute terminal illnesses.

### 2.4 Propensity score matching

To minimize selection bias and balance baseline characteristics between the groups, 1:1 propensity score matching was performed using a greedy nearest-neighbor algorithm without replacement. Propensity scores were estimated using logistic regression, with zinc deficiency status as the dependent variable. Covariates included demographics (age, sex, race/ethnicity, and body mass index), dementia subtype, comorbidities, laboratory parameters (e.g., estimated glomerular filtration rate, albumin, and C-reactive protein), and medication use (cardiovascular medications, antidepressants, antipsychotics, central nervous system medications, and antidiabetic agents). A caliper width of 0.1 standard deviations of the logit of the propensity score was applied. Covariate balance was assessed using standardized mean differences (SMDs), with values <0.1 indicating adequate balance. Propensity score density distributions were examined graphically to confirm sufficient overlap between the groups. The detailed diagnostic, procedural, laboratory, and medication codes used for cohort inclusion and exclusion, outcome definitions, and propensity score-matching variables are summarized in Supplementary Table 1.

### 2.5 Outcome assessment

The primary outcome was 1-year all-cause mortality. Secondary outcomes included sepsis (including severe sepsis), pneumonia, urinary tract infection, intensive care unit (ICU) admission, acute kidney injury (AKI), and elevated C-reactive protein levels (≥10 mg/L). Urinary tract infection was defined using ICD-10 code N39.0. Detailed diagnostic codes used to define study outcomes are summarized in Supplementary Table 1. Secondary outcomes were selected based on the established biological roles of zinc in immune function and inflammatory regulation. Zinc deficiency impairs both innate and adaptive immunity, potentially increasing susceptibility to infections, such as sepsis, pneumonia, and urinary tract infections, which are common complications in patients with dementia. Additionally, elevated C-reactive protein was assessed as a marker of systemic inflammation, given the evidence linking zinc deficiency to chronic low-grade inflammatory states that may accelerate disease progression and functional decline. Outcomes were defined using prespecified ICD codes and analyzed independently; they were not mutually exclusive, and patients could contribute to more than one outcome category if the corresponding diagnostic codes were present.

To avoid immortal time bias, the follow-up period commenced on day 31 after the index zinc measurement (landmark approach), ensuring that the initial 30-day exclusion window did not contribute to survival time calculations. Patients were followed up until the occurrence of each outcome, death, or the end of the follow-up period, whichever occurred first. To capture delayed complications, all outcomes were additionally evaluated during an extended follow-up window of 1–3 years.

### 2.6 Sensitivity, subgroup, and dose–response analyses

Two sensitivity analyses were performed to assess the robustness of the findings. Model I restricted analysis to patients with zinc measurements obtained between 2018 and 2024 to reflect contemporary clinical practice and reduce temporal heterogeneity. Model II restricted the analysis to patients aged ≥65 years, representing the predominant demographic affected by dementia with distinct nutritional and prognostic profiles. Subgroup analysis stratified by sex was conducted to explore potential effect modification, given the established biological differences in zinc metabolism between sexes. Interaction testing formally assessed whether the associations differed between male and female patients.

To evaluate the dose–response relationship, a separate analysis compared patients with severe zinc deficiency (<50 μg/dL) against those with normal zinc levels using identical matching procedures. The presence of a biological gradient, whereby more severe deficiency confers greater risk, would strengthen causal plausibility and help identify high-risk patients who may benefit the most from nutritional intervention.

### 2.7 Statistical analysis

Continuous variables were summarized as means with corresponding standard deviations, while categorical data were presented as counts and proportions. Consistent with the data structure of the TriNetX platform, analyses were conducted using an available-case approach to handle missing covariate information. Time-to-event outcomes were analyzed using Kaplan–Meier survival curves and Cox proportional hazards models. The follow-up commenced 30 days after the index date using a landmark design to reduce immortal time bias. Patients were censored at the time of outcome occurrence, death, or follow-up completion. Associations between the exposure groups and binary outcomes were estimated using hazard ratios with 95% confidence intervals. Statistical significance for the pre-specified primary outcome was defined using a two-sided *α* level of 0.05. As analyses of secondary outcomes were intended to be exploratory and hypothesis generating, adjustments for multiple comparisons were not applied. To evaluate the robustness of the primary findings to potential unmeasured confounding factors, E-values were calculated for both the point estimate and the limits of the confidence interval (22). All analyses were performed using built-in analytical modules provided within the TriNetX Research Network.

## 3 Results

### 3.1 Patient selection and baseline characteristics

**Figure 1:**
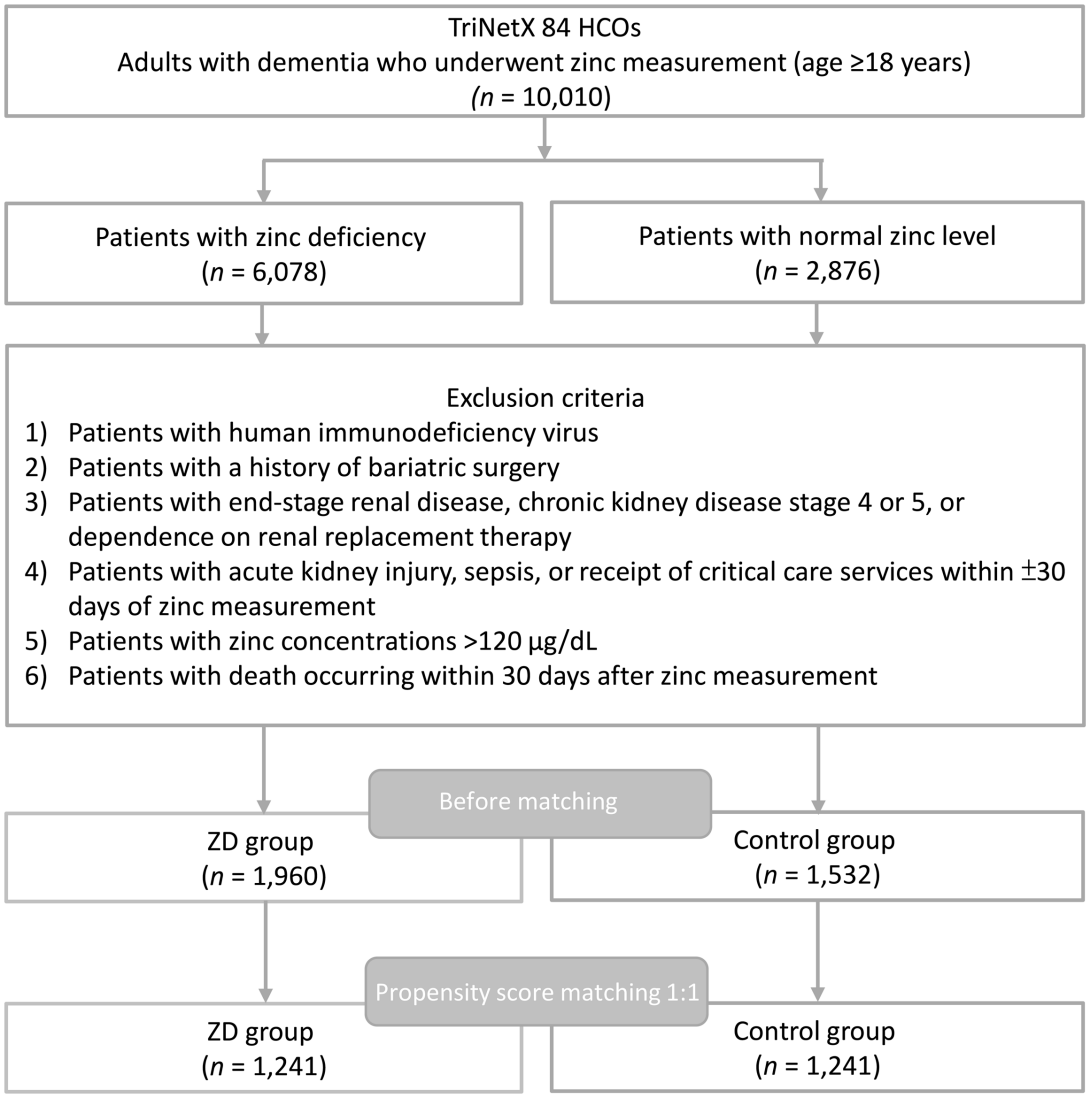
Flow diagram illustrating cohort selection from the TriNetX research network. HCOs, healthcare organizations; ZD, zinc deficiency.

From the TriNetX Research Network, encompassing 120 participating healthcare organizations, we identified 1,960 adult patients with dementia and zinc deficiency, and 1,532 patients with dementia and normal zinc levels who met all eligibility criteria (Figure 1). Following 1:1 propensity score matching, 1,241 patients remained in each cohort for subsequent analyses. Before matching, the zinc-deficient group was older than controls (mean age 75.1 ± 12.6 vs. 71.4 ± 15.0 years; median 77 vs. 75 years; interquartile range [IQR] 15 years in both groups). After 1:1 propensity score matching, age was well balanced (73.4 ± 13.6 vs. 73.6 ± 12.9 years; median 76 years in both cohorts; IQR 15 and 14 years), reflecting a predominantly older population. Patients with zinc deficiency were more frequently diagnosed with malnutrition (26.4% vs. 13.4%) and exhibited higher rates of elevated C-reactive protein (31.1% vs. 18.3%). Additional imbalances were observed in heart failure (22.2% vs. 15.6%), unspecified dementia (65.5% vs. 55.9%), and serum albumin ≥3.5 g/dL (74.2% vs. 82.8%) before matching (Table 1).

**Table 1:** Baseline characteristics of patients with dementia according to zinc status before and after propensity score matching.

Variables	Before matching			After matching		
	ZD group (*n* = 1,960)	Control group (*n* = 1,532)	SMD	ZD group (*n* = 1,241)	Control group (*n* = 1,241)	SMD
Age, years	75.1 ± 12.6	71.4 ± 15.0	0.270	73.4 ± 13.6	73.6 ± 12.9	0.016
Female	1,148 (58.6)	923 (60.2)	0.034	738 (59.5)	726 (58.5)	0.020
BMI > 30 kg/m^2^	444 (22.7)	416 (27.2)	0.104	337 (27.2)	323 (26.0)	0.026
White	1,203 (61.4)	1,055 (68.9)	0.158	835 (67.3)	818 (65.9)	0.029
Unknown race	375 (19.1)	209 (13.6)	0.149	189 (15.2)	199 (16.0)	0.022
Black or African American	278 (14.2)	181 (11.8)	0.070	150 (12.1)	155 (12.5)	0.012
Asian	36 (1.8)	28 (1.8)	0.001	22 (1.8)	24 (1.9)	0.012
Comorbidities						
Essential (primary) hypertension	1,263 (64.4)	973 (63.5)	0.019	815 (65.7)	811 (65.4)	0.007
Unspecified dementia	1,284 (65.5)	857 (55.9)	0.197	742 (59.8)	760 (61.2)	0.030
Dyslipidemia	978 (49.9)	865 (56.5)	0.132	691 (55.7)	687 (55.4)	0.006
Neoplasms	691 (35.3)	581 (37.9)	0.055	467 (37.6)	449 (36.2)	0.030
Dementia in other diseases classified elsewhere	607 (31.0)	560 (36.6)	0.118	437 (35.2)	431 (34.7)	0.010
Diabetes mellitus	630 (32.1)	485 (31.7)	0.010	402 (32.4)	405 (32.6)	0.005
Ischemic heart diseases	589 (30.1)	416 (27.2)	0.064	374 (30.1)	359 (28.9)	0.026
Disorders of thyroid gland	502 (25.6)	415 (27.1)	0.034	342 (27.6)	328 (26.4)	0.025
Vitamin D deficiency	457 (23.3)	414 (27.0)	0.085	336 (27.1)	324 (26.1)	0.022
Alzheimer’s disease	484 (24.7)	397 (25.9)	0.028	317 (25.5)	314 (25.3)	0.006
Chronic kidney disease (CKD)	369 (18.8)	254 (16.6)	0.059	230 (18.5)	219 (17.6)	0.023
Heart failure	435 (22.2)	239 (15.6)	0.169	226 (18.2)	223 (18.0)	0.006
Malnutrition	518 (26.4)	206 (13.4)	0.329	211 (17.0)	199 (16.0)	0.026
Overweight and obesity	263 (13.4)	260 (17.0)	0.099	205 (16.5)	194 (15.6)	0.024
Vascular dementia	301 (15.4)	249 (16.3)	0.025	198 (16.0)	199 (16.0)	0.002
Diseases of liver	295 (15.1)	179 (11.7)	0.099	168 (13.5)	158 (12.7)	0.024
COPD	272 (13.9)	171 (11.2)	0.082	158 (12.7)	154 (12.4)	0.010
Nicotine dependence	237 (12.1)	149 (9.7)	0.076	139 (11.2)	130 (10.5)	0.023
COVID-19	272 (13.9)	153 (10.0)	0.120	138 (11.1)	138 (11.1)	0.000
Respiratory failure	226 (11.5)	134 (8.7)	0.092	130 (10.5)	118 (9.5)	0.032
Alcohol-related disorders	208 (10.6)	117 (7.6)	0.103	102 (8.2)	111 (8.9)	0.026
Systemic connective tissue disorders	95 (4.8)	69 (4.5)	0.016	54 (4.4)	50 (4.0)	0.016
Laboratory data						
Hemoglobin ≥12 g/dL	1,473 (75.2)	1,242 (81.1)	0.143	995 (80.2)	982 (79.1)	0.026
Glomerular filtration rate ≥ 60 mL/min/1.73 m^2^	1,598 (81.5)	1,235 (80.6)	0.023	1,002 (80.7)	999 (80.5)	0.006
Albumin ≥3.5 g/dL	1,455 (74.2)	1,269 (82.8)	0.211	999 (80.5)	995 (80.2)	0.008
Hemoglobin A1c ≥ 7%	248 (12.7)	216 (14.1)	0.042	166 (13.4)	169 (13.6)	0.007
C reactive protein ≥10 mg/L	609 (31.1)	280 (18.3)	0.300	277 (22.3)	263 (21.2)	0.027
Medication						
Cardiovascular medications	1,661 (84.7)	1,224 (79.9)	0.127	1,036 (83.5)	1,020 (82.2)	0.034
Antidepressants	1,038 (53.0)	826 (53.9)	0.019	690 (55.6)	672 (54.2)	0.029
CNS medications	891 (45.5)	766 (50.0)	0.091	625 (50.4)	602 (48.5)	0.037
Antipsychotics	724 (36.9)	455 (29.7)	0.154	425 (34.2)	403 (32.5)	0.038
ACE inhibitors	443 (22.6)	307 (20.0)	0.063	282 (22.7)	259 (20.9)	0.045
Insulins and analogues	473 (24.1)	319 (20.8)	0.079	276 (22.2)	277 (22.3)	0.002
Angiotensin II inhibitor	411 (21.0)	279 (18.2)	0.070	237 (19.1)	243 (19.6)	0.012
Biguanides	223 (11.4)	206 (13.4)	0.063	157 (12.7)	150 (12.1)	0.017

Data are presented as mean ± standard deviation or *n* (%). BMI, body mass index; CKD, chronic kidney disease; CNS, central nervous system; COPD, chronic obstructive pulmonary disease; CRP, C-reactive protein; SMD, standardized mean difference; SMD <0.1 indicates adequate covariate balance between groups.

**Figure 2:**
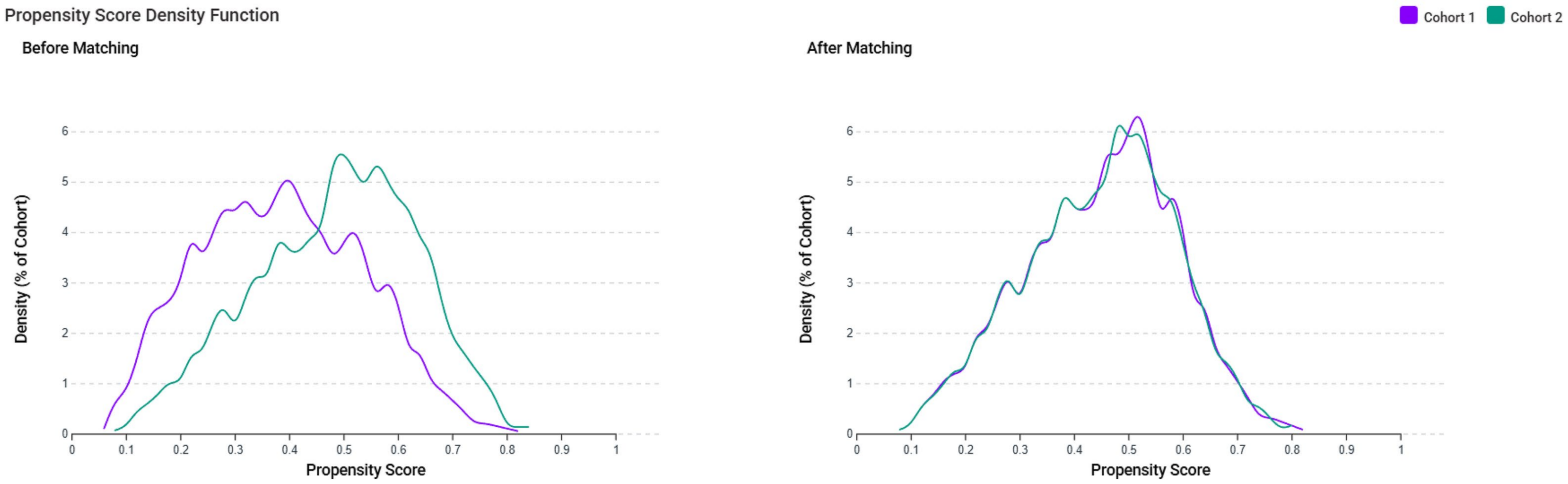
Propensity score distributions before and after matching. The figure displays the distribution of propensity scores for Cohort 1 (zinc deficiency; purple line) and Cohort 2 (control group; green line) before (left panel) and after (right panel) 1:1 propensity score matching. Before matching, the distributions show partial separation, indicating a baseline imbalance between cohorts. After matching, the marked overlap and nearly identical distributions demonstrated improved covariate balance and enhanced comparability between the two cohorts, supporting the robustness of subsequent outcome analyses.

After propensity score matching, all standardized mean differences fell below the prespecified threshold of 0.1, indicating an adequate covariate balance. The matched cohorts demonstrated comparable distributions for age (73.4 ± 13.6 vs. 73.6 ± 12.9 years), sex, race/ethnicity, dementia subtypes, comorbidities, laboratory parameters, and medication use. Propensity score density plots confirmed a substantial overlap between groups, supporting the validity of subsequent comparative analyses (Figure 2).

### 3.2 Association between zinc deficiency and 1-year outcomes

**Figure 3:**
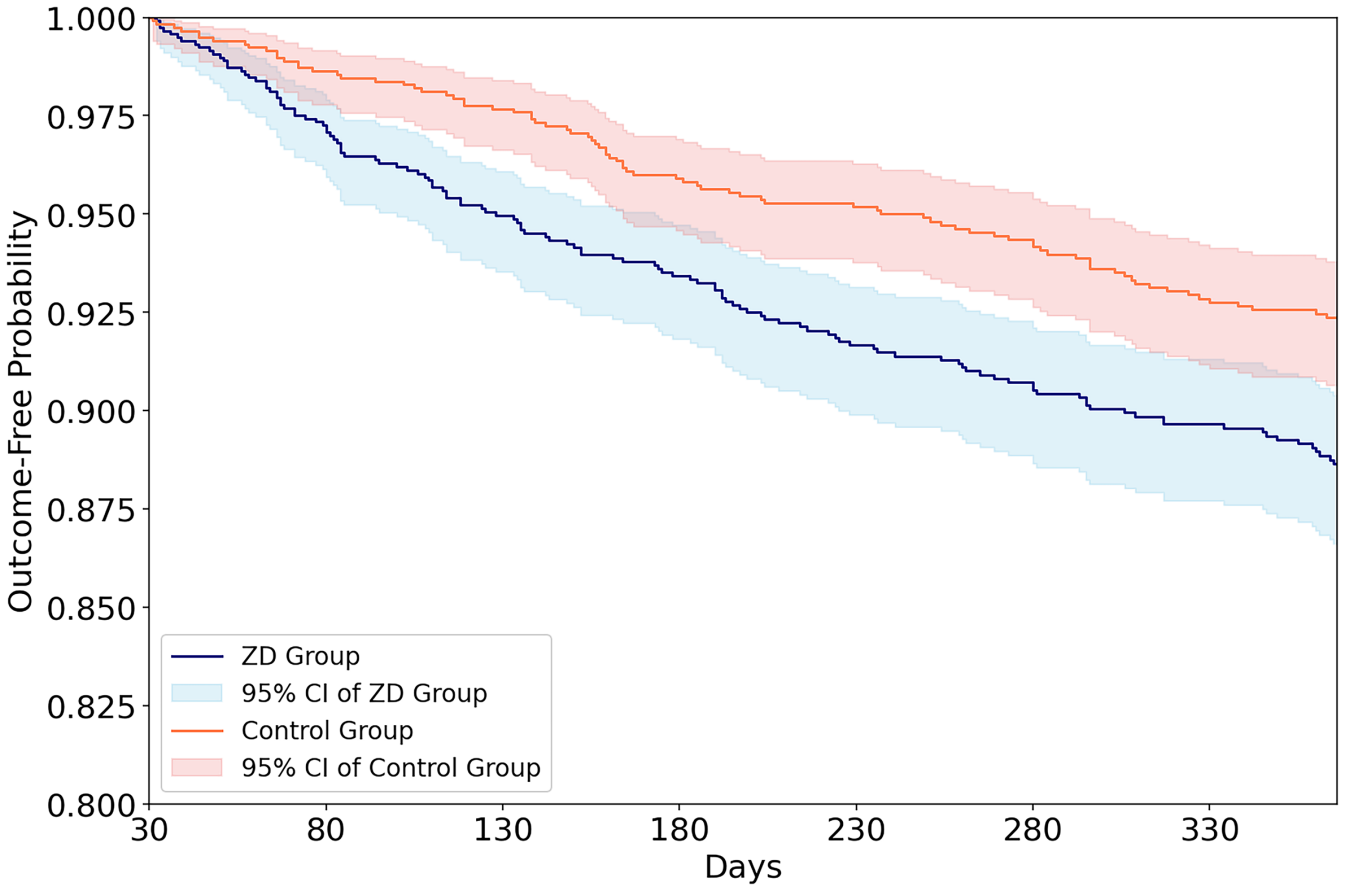
Outcome-free survival after landmark follow-up by zinc status. The figure shows outcome-free survival curves for patients with zinc deficiency (ZD group; blue line) and controls with normal zinc levels (orange line) beginning 30 days after the index zinc measurement (landmark analysis). Shaded areas represent 95% confidence intervals for each group. Across the follow-up period, patients in the ZD group exhibited a lower probability of remaining outcome-free compared with the control group, indicating an association between zinc deficiency and increased risk of adverse outcomes during follow-up.

During the first year of follow-up, commencing at day 31 after the index zinc measurement, patients with zinc deficiency experienced significantly higher all-cause mortality than controls (10.1% vs. 6.8%; HR 1.54; *p* = 0.002). Within the predefined 1-year follow-up window, the mean observed follow-up duration was 306.6 ± 110.7 days in the zinc-deficient group and 320.2 ± 100.9 days in the control group. The median follow-up duration was 365 days in both cohorts, consistent with the predefined 1-year landmark analysis. Kaplan–Meier survival curves demonstrated early and sustained separation between groups, with divergence becoming apparent within the first few months after cohort entry and persisting throughout the observation period (Figure 3).

Zinc deficiency was also associated with an increased risk of several secondary outcomes. The strongest association was observed for sepsis, where zinc-deficient patients exhibited a 76% higher risk than controls (HR 1.76; *p* = 0.001). Similarly, ICU admission occurred more frequently in the zinc-deficient cohort (HR, 1.67; *p* = 0.004). Urinary tract infections demonstrated a 47% elevated risk among zinc-deficient patients (HR, 1.47; *p* < 0.001), and elevated C-reactive protein levels were more common in this group (HR, 1.49; *p* = 0.002). In contrast, neither pneumonia (HR 1.10; *p* = 0.505) nor AKI (HR 1.28; *p* = 0.078) differed significantly between the cohorts during the 1-year follow-up period (Table 2).

**Table 2:** Association between zinc deficiency and clinical outcomes during 1-year follow-up (*n* = 1,241 for each group).

Outcomes	ZD group events (%)	Control group events (%)	HR (95% CI)	*p*-value (log-rank)
Mortality	125 (10.1)	85 (6.8)	1.54 (1.17–2.03)	0.002
Sepsis	85 (6.8)	51 (4.1)	1.76 (1.24–2.49)	0.001
ICU admission	81 (6.5)	51 (4.1)	1.67 (1.18–2.37)	0.004
Urinary tract infection	192 (15.5)	139 (11.2)	1.47 (1.18–1.83)	<0.001
Pneumonia	109 (8.8)	104 (8.4)	1.10 (0.84–1.43)	0.505
Acute kidney injury	112 (9.0)	92 (7.4)	1.28 (0.97–1.69)	0.078
Elevated CRP (≥10 mg/L)	148 (11.9)	105 (8.5)	1.49 (1.16–1.91)	0.002

CI, confidence interval; CRP, C-reactive protein; HR, hazard ratio; ICU, intensive care unit; ZD, zinc deficiency.

To evaluate the robustness of the primary outcome to potential unmeasured confounding, E-values were calculated. The E-value for the point estimate was 2.45, indicating that an unmeasured confounder would need to be associated with both zinc deficiency and mortality by a risk ratio of at least 2.45-fold to fully explain the observed association. The E-value for the lower confidence interval bound was 1.62, suggesting that to shift the confidence interval to include the null, an unmeasured confounder would require associations of at least this magnitude with both exposure and outcome. These values indicate moderate robustness to unmeasured confounding factors.

### 3.3 Association between zinc deficiency and extended outcomes

During the extended follow-up window of 1–3 years, no statistically significant differences were detected between the groups for any outcome (Table 3). Mortality rates were comparable (8.2% vs. 9.0%; HR 1.06; *p* = 0.659), as were the rates of sepsis, ICU admission, urinary tract infection, pneumonia, AKI, and elevated C-reactive protein levels (all *p* > 0.05). These findings suggest that the prognostic impact of zinc deficiency is most pronounced within the first year following measurement, with attenuation of risk differences during long-term follow-up.

**Table 3:** Association between zinc deficiency and clinical outcomes during extended follow-up (1–3 years) (*n* = 1,241 for each group).

Outcomes	ZD group events (%)	Control group events (%)	HR (95% CI)	*p-*value
Mortality	102 (8.2)	112 (9.0)	1.06 (0.81–1.39)	0.659
Sepsis	56 (4.5)	67 (5.4)	0.97 (0.68–1.38)	0.854
ICU admission	54 (4.4)	80 (6.4)	0.78 (0.55–1.11)	0.162
Urinary tract infection	152 (12.2)	162 (13.1)	1.09 (0.87–1.36)	0.459
Pneumonia	103 (8.3)	109 (8.8)	1.09 (0.83–1.43)	0.533
Acute kidney injury	93 (7.5)	106 (8.5)	1.02 (0.77–1.35)	0.883
Elevated CRP (≥10 mg/L)	102 (8.2)	102 (8.2)	1.15 (0.87–1.51)	0.321

CI, confidence interval; CRP, C-reactive protein; HR, hazard ratio; ICU, intensive care unit. ZD, zinc deficiency.

### 3.4 Sensitivity analyses, subgroup analysis, and dose–response analysis

Sensitivity analyses confirmed the robustness of the primary findings. When analysis was restricted to patients with zinc measurements obtained between 2018 and 2024 (Model I; *n* = 1,072 per group), zinc deficiency remained significantly associated with 1-year mortality (HR 1.42; *p* = 0.019). The association persisted in Model II, which included only patients aged ≥ 65 years (*n* = 1,093 per group; HR, 1.42; *p* = 0.014). Both sensitivity models also demonstrated consistent associations between urinary tract infection and elevated C-reactive protein levels (Table 4).

**Table 4:** Sensitivity analyses for the association between zinc deficiency and 1-year outcomes.

Outcomes	Model I		Model II	
	HR (95% CI)	*p*-value	HR (95% CI)	*p*-value
Mortality	1.42 (1.06–1.91)	0.019	1.42 (1.07–1.89)	0.014
Sepsis	1.40 (0.96–2.04)	0.079	1.58 (1.11–2.26)	0.011
ICU admission	1.20 (0.83–1.75)	0.332	1.57 (1.08–2.28)	0.016
Urinary tract infection	1.61 (1.28–2.03)	<0.001	1.48 (1.19–1.85)	<0.001
Pneumonia	1.08 (0.81–1.44)	0.619	1.04 (0.79–1.38)	0.760
Acute kidney injury	1.40 (1.04–1.89)	0.027	1.29 (0.96–1.73)	0.093
Elevated CRP (≥10 mg/L)	1.42 (1.09–1.85)	0.008	1.47 (1.13–1.90)	0.004

CI, confidence interval; CRP, C-reactive protein; HR, hazard ratio; ICU, intensive care unit. Model I: restricted to patients with zinc measurements obtained between 2018 and 2024 (*n* = 1,072 per group). Model II: restricted to patients aged ≥65 years (*n* = 1,093 per group).

Subgroup analysis stratified by sex revealed that the mortality association was statistically significant among female patients (HR, 1.60; *p* = 0.019), but not among males (HR, 1.13; *p* = 0.549) (Table 5). However, formal interaction testing did not indicate a significant effect modification by sex (*p* for interaction = 0.245), suggesting that the observed differences may reflect limited statistical power within strata rather than true biological heterogeneity.

**Table 5:** Subgroup analysis of the association between zinc deficiency and 1-year outcomes stratified by sex.

Outcomes	Male (*n* = 479)		Female (*n* = 751)		*p* for interaction
	HR (95% CI)	*p*-value	HR (95% CI)	*p-*value	
Mortality	1.13 (0.76–1.68)	0.549	1.60 (1.08–2.37)	0.019	0.245
Sepsis	1.51 (0.93–2.46)	0.092	1.80 (1.11–2.92)	0.016	0.632
ICU admission	1.38 (0.82–2.30)	0.222	1.05 (0.65–1.72)	0.834	0.479
Urinary tract infection	1.40 (0.95–2.07)	0.087	1.29 (1.00–1.68)	0.054	0.742
Pneumonia	1.27 (0.86–1.88)	0.231	1.19 (0.82–1.74)	0.367	0.819
Acute kidney injury	0.93 (0.61–1.43)	0.738	1.41 (0.95–2.10)	0.086	0.183
Elevated CRP (≥10 mg/L)	1.53 (1.05–2.24)	0.025	1.58 (1.13–2.19)	0.007	0.902

CI, confidence interval; CRP, C-reactive protein; HR, hazard ratio; ICU, intensive care unit.

Dose–response analysis comparing patients with severe zinc deficiency (<50 μg/dL) with those with normal zinc levels (*n* = 441 per group) revealed a biological gradient consistent with causal plausibility (Table 6). Severely zinc-deficient patients had an 85% higher mortality risk (HR 1.85; *p* = 0.002). The association with sepsis was particularly striking, with a nearly three-fold elevated risk (HR 2.92; *p* < 0.001). Stronger associations were also observed for ICU admission (HR 2.07), urinary tract infection (HR 2.16), pneumonia (HR 1.65), and elevated C-reactive protein levels (HR 1.93), all reaching statistical significance.

**Table 6:** Association between severe zinc deficiency and 1-year outcomes (*n* = 441 for each group).

Outcomes	Severe ZD group events (%)	Control group events (%)	HR (95% CI)	*p-*value
Mortality	65 (14.7)	40 (9.1)	1.85 (1.25–2.74)	0.002
Sepsis	50 (11.3)	20 (4.5)	2.92 (1.74–4.91)	<0.001
ICU admission	38 (8.6)	21 (4.8)	2.07 (1.21–3.53)	0.006
Urinary tract infection	102 (23.1)	56 (12.7)	2.16 (1.56–3.00)	<0.001
Pneumonia	68 (15.4)	48 (10.9)	1.65 (1.14–2.38)	0.008
Acute kidney injury	42 (9.5)	34 (7.7)	1.41 (0.90–2.21)	0.136
Elevated CRP (≥10 mg/L)	94 (21.3)	56 (12.7)	1.93 (1.39–2.69)	<0.001

CI, confidence interval; CRP, C-reactive protein; HR, hazard ratio; ICU, intensive care unit. Severe zinc deficiency (ZD) was defined as serum zinc concentration <50 μg/dL; the control group included patients with zinc levels within the reference range (70–120 μg/dL).

### 3.5 Multivariable cox proportional hazards model

In the multivariable Cox regression model adjusted for potential confounders, zinc deficiency retained independent prognostic significance for 1-year mortality (HR 1.74; *p* < 0.001) (Table 7). Additional independent predictors of mortality included male sex (HR 1.34; *p* = 0.011), advanced age (HR 1.04 per year; *p* < 0.001), and heart failure (HR 1.41; *p* = 0.010). Essential hypertension, neoplasms, ischemic heart disease, diabetes mellitus, chronic kidney disease, vitamin D deficiency, obesity, alcohol-related disorders, and chronic obstructive pulmonary disease did not reach statistical significance in the adjusted model.

**Table 7:** Multivariable cox proportional hazards model for 1-year mortality.

Variable	HR (95% CI)	*p-*value
Cohort 1 vs. Cohort 2 membership	1.74 (1.36–2.21)	<0.001
Male sex	1.34 (1.07–1.68)	0.011
Age at index (per year)	1.04 (1.03–1.06)	<0.001
Essential (primary) hypertension	0.91 (0.71–1.18)	0.479
Neoplasms	1.22 (0.97–1.53)	0.083
Ischemic heart disease	1.10 (0.85–1.41)	0.472
Diabetes mellitus	1.12 (0.88–1.42)	0.352
Chronic kidney disease	1.22 (0.94–1.60)	0.142
Vitamin D deficiency	0.91 (0.70–1.19)	0.485
Heart failure	1.41 (1.09–1.84)	0.010
Overweight and obesity	0.94 (0.67–1.32)	0.732
Alcohol-related disorders	1.39 (0.96–2.01)	0.085
COPD	1.26 (0.94–1.69)	0.123

CI, confidence interval; COPD, chronic obstructive pulmonary disease; HR, hazard ratio. Cohort 1 represents patients with zinc deficiency (<70 μg/dL) and Cohort 2 represents patients with normal zinc levels (70–120 μg/dL).

## 4 Discussion

In this cohort study of patients with dementia, zinc deficiency was significantly associated with elevated 1-year all-cause mortality, with a 54% higher risk compared to patients with normal zinc levels. Secondary analyses revealed that zinc deficiency was also linked to increased risks of sepsis, ICU admission, urinary tract infection, and elevated C-reactive protein levels during the first year of follow-up. Notably, these associations were attenuated during the extended to 1-to-3-year follow-up period. Dose–response analysis demonstrated a biological gradient, with severe zinc deficiency (<50 μg/dL) exhibiting strong associations with most outcomes. Sensitivity analyses limited to specific time periods and age groups produced findings consistent with the primary analysis, whereas E-value calculations suggested moderate robustness against unmeasured confounding.

Zinc deficiency demonstrated a significant association with 1-year all-cause mortality in our dementia cohort, a finding that aligns with the established biological roles of zinc in maintaining physiological homeostasis. Zinc participates in numerous cellular processes essential for survival, including immune defense, antioxidant protection, and inflammatory regulation (23, 24). Therefore, the observed mortality association may reflect the cumulative impact of zinc-dependent pathway dysfunction in a population already burdened by neurodegenerative disease. However, alternative explanations for this association merit further consideration. Zinc deficiency may serve as a marker of broader nutritional inadequacy rather than an independent prognostic factor. Patients with low zinc levels often exhibit concurrent deficiencies in other essential micronutrients and macronutrients, reflecting an overall poor nutritional status that commonly accompanies advanced dementia. Additionally, zinc deficiency may indicate underlying disease severity, decreased oral intake due to dysphagia or appetite loss, malabsorption, or heightened metabolic demands associated with chronic illnesses. Thus, the observed association may partly reflect zinc status as a surrogate marker for general health decline or frailty rather than a direct mechanistic contributor to mortality.

In the current study, this finding is novel in specifically characterizing the prognostic significance of zinc status within the dementia population. While previous studies have documented associations between zinc deficiency and mortality in critically ill and hospitalized patients (18–20), direct evidence in dementia has been limited. Our results extend these observations to patients with dementia, suggesting that zinc assessment may provide clinically relevant prognostic information for this vulnerable population. The observation that prognostic significance was limited to the 1-year window, with diminishing associations during longer follow-up, implies that zinc deficiency may mark an acute or reversible state of nutritional or physiological stress rather than a persistent determinant of long-term outcomes. From a clinical perspective, this time-dependent association supports the use of zinc assessment to identify individuals at increased short-term risk who may warrant targeted intervention or enhanced surveillance.

Dose–response analysis strengthens the plausibility of our findings by demonstrating a biological gradient. When comparing patients with more severe zinc deficiency (<50 μg/dL) with those with normal zinc levels, the mortality association was more pronounced (HR 1.85) than that observed in the primary analysis using the conventional deficiency threshold of <70 μg/dL (HR 1.54). Similarly, amplified associations were observed for sepsis, ICU admission, and urinary tract infection. This gradient, whereby lower zinc concentrations correspond to greater risk, suggests that the severity of zinc depletion correlates with the magnitude of the risk of adverse outcomes, a pattern consistent with biological plausibility. The E-value of 2.45 for the mortality point estimate indicates that an unmeasured confounder would need associations of at least this magnitude with both zinc deficiency and mortality to fully explain the observed relationship. While this does not exclude confounding factors, it suggests that modest unmeasured factors are unlikely to completely account for the findings.

Our results revealed that zinc deficiency was associated with significantly elevated risks of sepsis and urinary tract infection during a 1-year follow-up, consistent with the established biological roles of zinc in immune function. The particularly strong association with sepsis (HR 1.76) is noteworthy, given the high mortality burden of sepsis in patients with dementia (2, 25). Prior research by Hoeger et al. (19) documented links between low serum zinc levels and recurrent septic episodes in critically ill patients, and our observations extend this relationship to the dementia population. Dose–response analysis revealed an even more pronounced association with severe zinc deficiency (HR, 2.92), further supporting this relationship. In contrast, pneumonia showed no significant association with zinc deficiency in the primary analysis, although severe deficiency demonstrated an elevated risk of pneumonia (HR 1.65). This differential pattern may reflect the multifactorial etiology of pneumonia in patients with dementia, where aspiration risk and impaired airway protection may predominate over immune dysfunction as primary determinants.

The association between zinc deficiency and elevated C-reactive protein levels (HR, 1.49) in our cohort suggests a link with systemic inflammatory activity. This finding is supported by interventional and observational evidence that zinc status is closely related to inflammatory and oxidative stress markers. A systematic review and meta-analysis of randomized controlled trials showed that zinc supplementation significantly reduced serum concentrations of C-reactive protein, tumor necrosis factor-*α*, and markers of oxidative stress, indicating that inadequate zinc availability contributes to a pro-inflammatory and pro-oxidative milieu (26). Consistent with these findings, observational data from patients with chronic liver disease demonstrated inverse associations between serum zinc levels and C-reactive protein, nitric oxide, and malondialdehyde levels, further supporting the relationship between zinc deficiency, inflammation, and oxidative stress in clinical populations (27). In patients with dementia, chronic low-grade inflammation has been implicated in disease progression, functional decline, and increased vulnerability to medical complications (28, 29). Therefore, the observed association between zinc deficiency and elevated inflammatory markers in our study may reflect shared inflammatory and oxidative pathways or may identify a subgroup of patients with heightened systemic inflammatory burden who face increased short-term risk. Notably, this relationship remained robust across sensitivity analyses and exhibited dose–response characteristics, supporting its potential clinical relevance.

Several limitations warrant consideration when interpreting these findings. First, because this analysis was observational, causal relationships could not be established, and residual confounding factors not captured in the dataset may persist. The TriNetX database does not provide detailed information on socioeconomic status (e.g., income), dietary intake, caloric consumption, or living assistance status; therefore, residual confounding related to unmeasured nutritional and social determinants of health cannot be fully excluded despite adjustment for multiple demographic, comorbidity, laboratory, and medication-related proxies. Second, zinc measurement in clinical practice is not performed randomly; patients undergoing testing may represent a selected population with specific clinical indications (e.g., suspected nutritional deficiency or malabsorption), potentially limiting the generalizability to unselected dementia populations. Therefore, our findings may primarily reflect patients who underwent clinical zinc assessment rather than an unselected dementia population. In addition, zinc and copper share intestinal absorption pathways and exhibit biological antagonism. However, serum copper measurements were not routinely available in the TriNetX database, and the interplay between zinc and copper status could not be directly assessed. Third, single zinc measurements may not adequately capture dynamic changes in nutritional status over time or account for physiological fluctuations. It is possible that changes in zinc status over time may have influenced extended (1–3 years) outcomes and contributed to the attenuation of associations observed during longer follow-up. Accordingly, our findings primarily reflect the prognostic relevance of baseline zinc status rather than dynamic zinc trajectories. Future studies incorporating longitudinal zinc measurements are warranted to clarify the impact of temporal zinc variations on long-term outcomes. Fourth, the TriNetX database lacks granular information regarding dementia severity, cognitive function, functional status, and dietary intake, all of which may independently influence both zinc status and clinical outcomes. Fifth, despite the large multicenter design encompassing diverse healthcare organizations, the findings may not generalize to populations outside the United States healthcare systems or to patients not captured in electronic health records. Finally, the available-case approach for handling missing covariate information may introduce bias if the data are not missing completely at random.

## 5 Conclusion

In this large matched cohort study, zinc deficiency was associated with increased 1-year mortality, with evidence of a dose–response relationship. These associations were attenuated during extended follow-up, suggesting prognostic relevance primarily within the first year. Although these findings do not establish causation, they suggest that zinc status may serve as a clinically useful prognostic marker in this population. Future prospective studies should investigate whether zinc supplementation modifies outcomes in zinc-deficient dementia patients and explore the temporal dynamics of zinc status in relation to disease progression.

## Data Availability

The raw data supporting the conclusions of this article will be made available by the authors, without undue reservation.
