# Supplementary material for: Association between zinc deficiency and adverse outcomes in patients with dementia: a matched cohort study
Source: Front Nutr. 2026 Mar 20;13:1801558. doi: 10.3389/fnut.2026.1801558 (PMC13046538; doi:10.3389/fnut.2026.1801558)
Supplement: Supplementary file 1 [file Table_1.DOCX]

**Supplemental Table 1. Codes Used for Cohort Definition, Outcomes, and Propensity Score Matching**

| Category | Variable | Code(s) / Definition |
| --- | --- | --- |
| Inclusion Criteria | Age | ≥18 years |
|  | Dementia (any) | F01 (Vascular dementia); F02 (Dementia in other diseases); F03 (Unspecified dementia); G30 (Alzheimer’s disease) |
|  | Zinc deficiency (ZD cohort) | 5763-8, Zinc [Mass/volume] in serum or plasma <70 µg/dL |
|  | Normal zinc (Control cohort) | 5763-8, Zinc [Mass/volume] in serum or plasma 70–120 µg/dL |
| Exclusion Criteria | HIV infection | B20 |
|  | Bariatric surgery status | Z98.84 |
|  | End-stage renal disease | N18.6 |
|  | Advanced CKD | N18.4, N18.5 |
|  | Dialysis dependence | Z99.2 |
|  | Acute kidney injury (±1 month of zinc test) | N17 |
|  | Sepsis (±1 month of zinc test) | A41, R65.2 |
|  | Critical care services (±1 month of zinc test) | 1013729 |
|  | Death within 1 month after zinc test | Deceased; R99 |
| Primary Outcome | All-cause mortality | Deceased; R99 |
| Secondary Outcomes | ICU admission | 1013729 |
|  | Sepsis | R65.2; A41 |
|  | Pneumonia | J12–J18; J69; ICD-9-CM 483 |
|  | Urinary tract infection | N39.0 |
|  | Acute kidney injury | N17 |
|  | Elevated C-reactive protein | 9063, CRP ≥10 mg/L (most recent value) |
| Propensity Score Matching Variables | Demographics | Age, sex, race/ethnicity, body mass index |
|  | Dementia subtype | F01, F02, F03, G30 |
|  | Cardiovascular comorbidities | I10, I20–I25, I50 |
|  | Metabolic disorders | E08–E13 (diabetes), E66 (obesity), E78 (dyslipidemia) |
|  | Renal disease | N18 |
|  | Pulmonary disease | J44 |
|  | Liver disease | K70–K77 |
|  | Malnutrition | E40–E46 |
|  | Other comorbidities | F17 (smoking), F10 (alcohol), E55 (vitamin D deficiency), C00–D49 (neoplasms), U07.1 (COVID-19) |
|  | Medications | Insulin (A10A), Biguanides (A10BA), ACE inhibitors (CV800), ARBs (CV805), cardiovascular medications (CV000), CNS medications (CN900), antipsychotics (CN700), antidepressants (CN600) |
|  | Laboratory variables | Hemoglobin (9014), Albumin (9045), eGFR CKD-EPI (98979-8), CRP (9063), HbA1c (9037), BMI (9083) |
